# *PNPLA3* Polymorphism Is Inversely Correlated with Aortic Stiffness in Patients with Metabolic Dysfunction-Associated Steatotic Liver Disease Without Fibrosis

**DOI:** 10.3390/ijms26073256

**Published:** 2025-04-01

**Authors:** Barbara Toffoli, Consuelo Comar, Andrea Grillo, Vincenzo Barbato, Emanuele Vincis, Veronica Baldi, Silvia Berti, Teresa Volpato, Francesca Zorat, Saveria Lory Crocè, Giacomo Emmi, Bruno Fabris, Massimo Puato, Stella Bernardi

**Affiliations:** 1 Department of Medical Surgical and Health Sciences, University of Trieste, Cattinara Teaching Hospital Strada di Fiume, 34100 Trieste, Italy; andrea.grillo@units.it (A.G.); barbato.vinc@gmail.com (V.B.); emanuele.vincis@studenti.units.it (E.V.); veronica.baldi@studenti.units.it (V.B.); silvia.berti@studenti.units.it (S.B.); lcroce@units.it (S.L.C.); giacomo.emmi@units.it (G.E.); b.fabris@fmc.units.it (B.F.); massimo.puato@units.it (M.P.); stella.bernardi@units.it (S.B.); 2 UCO Medicina Clinica ASUGI, Cattinara Teaching Hospital, Strada di Fiume, 34100 Trieste, Italy; consuelo.comar@asugi.sanita.fvg.it (C.C.); francesca.zorat@asugi.sanita.fvg.it (F.Z.); 3 Centro Clinico Studi Fegato ASUGI, Maggiore Teaching Hospital, Piazza dell’Ospitale, 34100 Trieste, Italy; 4 SSD Angiologia ASUGI, Cattinara Teaching Hospital, Strada di Fiume, 34100 Trieste, Italy; 5 SS Endocrinologia ASUGI, Cattinara Teaching Hospital, Strada di Fiume, 34100 Trieste, Italy

**Keywords:** *PNPLA3*, MASLD, CVD, cardiovascular risk, aortic stiffness, arterial tonometry, carotid intima-media thickness

## Abstract

Metabolic dysfunction-associated steatotic liver disease (MASLD) corresponds to the condition of increased hepatic fat levels, which is the leading cause of hepatic failure and carcinoma. It is also an independent risk factor for cardiovascular disease (CVD) and mortality. MASLD can be due to obesity with insulin resistance and/or genetic predisposition, i.e., polymorphism in the patatin-like phospholipase domain-containing 3 (*PNPLA3*) gene. *PNPLA3* polymorphism has been associated with increased hepatic fat levels, fibrosis, cirrhosis, and hepatocellular carcinoma, while its association with CVD remains to be fully understood. The aim of the current study was to examine whether the vascular phenotype of patients with MASLD differed between carriers and noncarriers of the *PNPLA3* polymorphism. Adult patients with MASLD underwent clinical assessment, *PNPLA3* genotyping, arterial tonometry for aortic stiffness measurement, and ultrasound examination of carotid arteries. In total, 117 patients with MASLD and no fibrosis (median hepatic stiffness was 4.71 kPa) were recruited. Carriers of the *PNPLA3* polymorphism were younger and exhibited higher levels of ALT and APRI, as compared to wild-type subjects. On the other hand, carriers of the *PNPLA3* polymorphism had not only a better metabolic profile (i.e., lower glucose and glycated hemoglobin) but also lower blood pressure, carotid intima-media thickness (IMT), and cardiovascular risk. In addition, *PNPLA3* polymorphism was negatively correlated with aortic stiffness, which is a marker of arteriolosclerosis and vascular ageing. Our data are consistent with previous observations that in case of genetically-driven MASLD, there is an inverse association with common predictors of CVD. Our data support the view that the main contributors to CVD risk in patients with MASLD remain conventional cardiometabolic risk factors (i.e., age, glucose) that are more likely to be found in metabolic syndrome-related MASLD rather than genetically-driven MASLD, at least in the first stages of the disease.

## 1. Introduction

Liver steatosis has become the most common cause of chronic liver disease worldwide and is projected to become the leading cause of hepatocellular carcinoma and end-stage liver disease. In addition, substantial epidemiological evidence from large cohort studies indicate that liver steatosis is also an independent risk factor for cardiovascular disease (CVD) morbidity and mortality [1].

Recently, the nomenclature of liver steatosis has changed. The term metabolic dysfunction-associated steatotic liver disease (MASLD) has replaced the word nonalcoholic fatty liver disease (NAFLD) [2]. The rationale behind this new fatty liver disease nomenclature was that the more specific term MASLD provides an affirmative non-stigmatizing metabolic descriptor of the condition rather than a diagnosis of exclusion, as “fatty” and “nonalcoholic” are replaced by “steatotic” and “metabolic dysfunction”. In parallel, the way to diagnose this condition has also been reviewed, such that today the diagnosis of MASLD should be made in case of hepatic steatosis and at least one cardiometabolic risk factor in the absence of other causes of liver disease. Cardiometabolic risk factors, which are intended to identify patients with insulin resistance, include overweight, altered glucose metabolism, high blood pressure, high triglycerides, and low HDL cholesterol. This diagnostic approach was chosen as being straightforward and easy to apply in clinical practice. In addition, it appears that 98% of the existing registry cohort of patients with NAFLD would fulfill the new criteria for MASLD [2].

The development and progression of MASLD has a strong genetic component, involving mostly genes contributing to hepatic lipid processing. The most robust of these associations is with a single nucleotide polymorphism of the patatin-like phospholipase domain-containing 3 (*PNPLA3*) gene, as the G allele in *PNPLA3* (rs738409[G]), encoding I148M protein, was strongly correlated with increased hepatic fat levels [3]. Interestingly, in the multisociety Delphi consensus statement on fatty liver nomenclature, polymorphisms of genes involved in hepatic lipid processing, such as the *PNPLA3* gene, that are common in the general population, were not considered a distinct nosological entity, but disease modifiers of MASLD. Therefore, it has been argued that MASLD might have at least two distinct phenotypes [4]. On one hand, it may affect patients with the metabolic syndrome phenotype, where liver steatosis is the result of obesity with hepatic insulin resistance, which impairs the ability of insulin to inhibit glucose and VLDL production. On the other hand, it may affect patients without the metabolic syndrome phenotype, but with genetic predisposition, where liver steatosis is due to genetic impairment of lipolysis, such as in the presence of genetic variation of *PNPLA3* gene.

Genetic variant in *PNPLA3* has been associated not only with liver fat accumulation but also with susceptibility to steatohepatitis, fibrosis, cirrhosis, and hepatocellular carcinoma [5,6]. By contrast, it remains to be elucidated whether there is an independent association between the *PNPLA3* genotype and CVD morbidity and mortality [7]. For instance, although convincing evidence now substantiates a link between MASLD and early carotid atherosclerosis [8], the few observational studies that have evaluated the association between *PNPLA3* gene variants and early carotid atherosclerosis have provided conflicting results [7,9,10,11].

Based on these premises, the aim of the current study was to examine whether the vascular phenotype (i.e., aortic stiffness and carotid intima-media thickness) of patients with MASLD differed between carriers and noncarriers of the *PNPLA3* polymorphism, rs738409[G] allele.

## 2. Results

### 2.1. MASLD Patients’ Characteristics

The clinical characteristics of the MASLD patients that we recruited are shown in Table 1. All the patients received the diagnosis of MASLD based on the multisociety Delphi consensus statement [2]. They were on average 63 years old, overweight (median BMI 28.5 Kg/m^2^), and with altered glucose metabolism (fasting glucose 106 mg/dL). A total of 36% of them had type 2 diabetes mellitus. Focusing on the liver disease, median hepatic stiffness as assessed by ultrasound elastography, was 4.71 kPa, indicating no fibrosis. This result was consistent with noninvasive scores as it correlated with APRI (rho = 0.27, *p* = 0.02) and FIB-4 (rho = 0.21, *p* = 0.07).

### 2.2. Observed and Reported Allele Frequencies of the Minor G Allele in PNPLA3 rs738409

In our cohort of patients (*n* = 117), *PNPLA3* rs738409 genotype distribution was as follows: CC, *n* = 37 (31.6%); GC, *n* = 59 (50.4%), and GG, *n* = 21 (18.0%), as shown in Table 2. We found a deviation from the Hardy–Weinberg equilibrium (*p* < 0.0001), when we compared the observed G allele frequency in our cohort to its reported frequency in a control Caucasian (European) population (publicly available from https://www.ncbi.nlm.nih.gov/snp/rs738409, accessed on 10 November 2024) [12], indicating that the risk of disease *per se* may be influenced by the polymorphism [13]. Consistent with this, the G allele frequency observed in our study was similar to that reported by other authors in patients with MASLD (*p* = 0.328) [14].

### 2.3. Metabolic Differences Between Patients with and Without PNPLA3 rs738409[G] Allele

**Figure 1:**
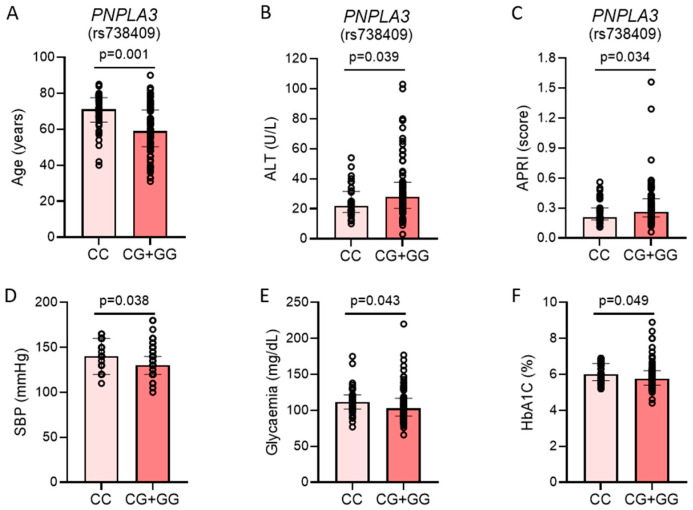
Metabolic differences between patients with and without *PNPLA3* rs738409[G] allele. Variations in (**A**) age; (**B**) alanine aminotransferase (ALT); (**C**) aspartate aminotransferase to platelet ratio index (APRI); (**D**) systolic blood pressure (SBP); (**E**) glycaemia; (**F**) glycated hemoglobin (HbA1c). Results are reported as median (IQR). Comparison within groups was performed with the Wilcoxon test. *p* < 0.05 was considered statistically significant.

In our cohort of patients, both groups were overweight and they had steatosis with no fibrosis (Table 1). Carriers of the *PNPLA3* rs738409[G] allele were younger but exhibited higher levels of ALT and APRI (Figure 1). On the other hand, patients without the *PNPLA3* polymorphism had more signs of metabolic syndrome. In particular, they exhibited higher systolic blood pressure, higher fasting glucose, and higher glycated hemoglobin (Figure 1). The percentage of subjects with type 2 diabetes mellitus did not differ between the two groups, nor the percentage of patients taking metformin, SGLT2i, or GLP-1RA. On the other hand, more patients without the *PNPLA3* polymorphism were taking lipid-lowering drugs, such that they had lower LDL cholesterol (Table 1).

### 2.4. Vascular Differences Between Patients with and Without PNPLA3 rs738409[G] Allele

**Figure 2:**
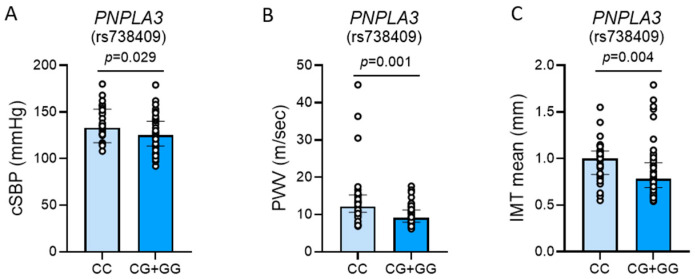
Cardiovascular phenotype of patients with and without *PNPLA3* rs738409[G] allele. (**A**) central systolic blood pressure (cSBP); (**B**) pulse wave velocity (PWV); (**C**) mean intima-media thickness (IMT mean). Results are reported as median +IQR. Comparison within groups was performed with the *t*-test or the Wilcoxon test based on data distribution. *p* < 0.05 was considered statistically significant.

Table 3 and Figure 2 show the vascular differences between patients with and without the *PNPLA3* rs738409[G] allele. Patients with the *PNPLA3* polymorphism exhibited lower systolic blood pressure and pulse pressure (PP). In particular, PP is the difference between systolic and diastolic blood pressure and the higher it is, the higher is the stiffness of large vessels and the cardiovascular risk [15,16]. Arterial tonometry confirmed that patients with the *PNPLA3* polymorphism had lower systolic blood pressure at the aortic level (i.e., cSBP), and lower systolic slope, which is the rate of rise of arterial blood pressure during the early systolic phase [15,16,17]. In addition, arterial tonometry showed that these patients had lower pulse wave velocity (PWV). The measurement of PWV is considered the gold standard for assessing aortic stiffness [18,19], which is a marker of arteriolosclerosis and vascular aging [20]. Consistent with these data, carotid ultrasound showed that patients with the *PNPLA3* polymorphism had also lower intima-media thickness (IMT), which is a marker of early-stage atherosclerosis that predicts CVD risk [20].

### 2.5. Multivariate Regression Analysis

Multivariate linear regression analyses (Table 4) showed that cSBP and PP were independently associated with age (*p* = 0.03 and *p* < 0.001). PWV was independently associated with age (*p* < 0.001), but it was also inversely related to *PNPLA3* polymorphism (*p* = 0.02). As for carotid IMT, this was associated with age and male sex (*p* < 0.001 and *p* = 0.02).

## 3. Discussion

In this study, we sought to examine whether the vascular phenotype of patients with MASLD differed between carriers and noncarriers of the *PNPLA3* polymorphism. In line with the European Society of Hypertension recommendations, we looked at aortic stiffness and carotid IMT, as markers of vascular damage and predictors of CVD [20]. Among 117 patients with MASLD without evidence of fibrosis, 68.4% of the subjects were carriers of the minor G allele in *PNPLA3* rs738409. The G allele in *PNPLA3* rs738409 encodes for a protein variant (pI148M) with loss-of-function of triglyceride hydrolase and trans-acylase activity in lipid droplets, leading to accumulation of polyunsaturated fatty acids, and reduction of secretion of VLDL from hepatocytes [21]. Our data show that carriers of the G allele were younger and exhibited higher levels of ALT and APRI, as compared to wild-type subjects (homozygous for the C allele). This is consistent with the observation that genetic variation in *PNPLA3* confers susceptibility to hepatic fat content and liver injury [3], and it is associated with an earlier age of liver disease diagnosis [22].

Looking at the metabolic phenotype of these patients, carriers of the G allele exhibited significantly lower glucose and glycated hemoglobin levels as well as lower HOMA index, as compared to subjects homozygous for the C allele. This is consistent with the concept that MASLD represents a heterogeneous condition showing a wide spectrum of clinical and pathophysiological sub-phenotypes [4,23]. In particular, it is current scientific opinion that there are at least two distinct forms of MASLD: one that is acquired and it is due to metabolic dysfunction, the other that is genetic and it is due to single-nucleotide polymorphisms leading to hepatic lipotoxicity [4]. In the first case, MASLD stems from visceral adiposity leading to hepatic insulin resistance, which impairs the ability of insulin to inhibit hepatic glucose and VLDL production. This leads to hyperglycemia, hyperinsulinemia, hypertriglyceremia, and a low HDL concentration that are associated with liver steatosis. In the second case, gene variants, like the *PNPLA3* polymorphism, may cause lipid alteration in hepatocytes, such as in droplet retention or VLDL secretion, impaired synthesis or catabolism determining lipotoxicity. This leads to liver steatosis, without hyperglycemia and hyperinsulinemia at least in the first stages of the disease.

Patients with MASLD have a two-fold increased risk for CVD [8]. When looking at carotid atherosclerosis and carotid IMT, MASLD as defined by fatty liver index has been found associated with the presence and progression of carotid IMT [24]. More recently, Zhou et al. showed that MASLD was associated not only with increased carotid IMT/plaques [OR 1.74; 95%CI (1.47–2.06)], but also with increased arterial stiffness [OR 1.56; 95%CI (1.24–1.96)], coronary artery calcification [OR 1.40; 95%CI (1.22–1.60)], and endothelial dysfunction [OR 3.73; 95%CI (0.99–14.1)] [25]. In addition, when looking at hard clinical outcomes, Targher et al. found that MASLD patients had a higher prevalence of coronary (26.6% vs. 18.3%), cerebrovascular (20% vs. 13.3%), and peripheral vascular disease (15.4% vs. 10%) than those without this condition [26]. Consistent with these data, a large meta-analysis, including 36 longitudinal studies with a median follow-up of 6.5 years, 5.8 million people, and 99,668 cases of CVD showed that MASLD was associated with a moderately increased risk of CVD events (HR: 1.45 95%CI 1.31–1.61) and this risk increased in parallel with the severity of MASLD [27].

However, the risk for CVD may differ between the metabolic syndrome-related MASLD and the genetically-driven MASLD [7,28]. Although Petta et al. found that the *PNPLA3* G allele was associated with higher severity of carotid atherosclerosis [9], recent studies have shown either no correlation between three MASLD-associated polymorphisms and subclinical atherosclerosis [11], or an inverse correlation between them [10]. In addition, a few studies have also shown that the *PNPLA3* G allele was inversely related to cardiovascular risk or CVD [29,30,31]. In particular, Ruschenbaum et al. reported that the *PNPLA3* G allele was associated with liver disease but with a relatively benign cardiovascular risk profile [31]. Additionally, Wu et al. showed that patients with the G allele in *PNPLA3* had a lower risk of coronary disease [OR 0.6; 95%CI(0.4–0.9)] [29]. Likewise, Akuta et al. found that the absence of *PNPLA3* polymorphism was independently associated with the risk of CVD in patients with MASLD [HR 3.66; 95%CI (1.63–8.35)] [30]. They argued that patients with the *PNPLA3* CC genotype appeared to have higher fasting plasma glucose and rates of type 2 diabetes mellitus, which might explain their higher CVD risk [30]. Consistent with this view, our data show that carriers of the G allele in *PNPLA3* not only had a better metabolic profile (i.e., lower glucose and glycated hemoglobin) but also lower blood pressure, pulse pressure, cSBP, aortic stiffness, IMT, and cardiovascular risk, as compared to patients with metabolic syndrome-related steatotic liver disease.

Interestingly, our study shows for the first time that carriers of the G allele in *PNPLA3* had lower aortic stiffness, as assessed by arterial tonometry, and that this association was independent of patients’ age, sex, and glucose levels. Aortic stiffness is a marker of arteriolosclerosis and vascular aging [20] and it represents a validated predictor of CVD morbidity and mortality [32,33], which allows cardiovascular risk to be reclassified in several clinical situations and which seems to be particularly useful in patients at intermediate risk as well as in patients without standard cardiovascular risk factors [34,35]. Previous studies found that in patients with MASLD, aortic stiffness was correlated with liver fibrosis, and that this correlation was stronger among patients with diabetes and metabolic syndrome [36]. However, this is the first study differentiating aortic stiffness between patients with metabolically, versus genetically driven, MASLD, and here we show that aortic stiffness (i.e., arteriosclerosis) is increased in metabolically driven MASLD.

As for carotid IMT, which is a marker of early atherosclerosis and a validated predictor of CVD morbidity and mortality [20], our data show that subjects with the *PNPLA3* G allele had lower IMT than patients with the CC genotype, being 0.78 mm vs. 1.00 mm respectively. Although in our work the *PNPLA3* genotype was not independently related to IMT, our data are in line with an earlier study by Di Costanzo et al. [10] who found that median carotid IMT in the group of metabolically driven MASLD was significantly higher than that in the group of genetically driven MASLD, being 0.85 mm vs. 0.66 mm respectively. In other words, our data support the view that “Not all forms of MASLD were created equal” [7] and that metabolic syndrome-related MASLD has a greater impact on the aortic stiffening and early carotid atherosclerosis as compared to the *PNPLA3*-related form.

MASLD is a heterogeneous entity. For this reason, one study limitation could be the intrinsic difference in the phenotype of metabolic syndrome-driven MASLD vs. genetic MASLD (e.g., age and glucose). Otherwise, limitations of this study include the Mediterranean background of the patients, which might have an impact on genotype distribution [12] as well as lifestyle, and the early stage of MASLD. Nevertheless, this study shows for the first time that *PNPLA3* polymorphism was inversely correlated with aortic stiffness in patients with MASLD without fibrosis. Our data support the view that the main contributors to CVD risk in patients with MASLD remain conventional cardiometabolic risk factors (i.e., age, glucose) that are more likely to be found in metabolic syndrome-related MASLD rather than genetically driven MASLD, at least in the first stages of the disease. These data might help differentiate patient follow-up, prioritizing CVD assessment in metabolic syndrome-related MASLD. Further studies are needed to confirm and extend our findings to more advanced stages of the disease.

## 4. Materials and Methods

### 4.1. Study Design

This is an observational cross-sectional study including adult patients (>18 years) with MASLD, as defined by Rinella et al. [2]. In the presence of hepatic steatosis, the finding of any cardio-metabolic risk factor (obesity, altered glucose metabolism, blood pressure ≥130/85 mmHg or antihypertensive drug treatment, hypertriglyceridemia or lipid-lowering treatment, low plasma HDL cholesterol or lipid-lowering treatment) would confer a diagnosis of MASLD if there were no other causes of hepatic steatosis.

Patients were consecutively selected in the outpatient Hepatology Service of Cattinara Teaching Hospital (Ambulatorio Epatologico UCO Medicina Clinica, ASUGI) between November 2023 and December 2024. Patients were included after providing informed consent to participate in this study. After enrollment, patients underwent full clinical assessment, venous blood sampling at fasting for general biochemistries and genotyping, as well as arterial tonometry and ultrasound measurement of carotid intima-media thickness.

### 4.2. Clinical Assessment, General Biochemistries, and Noninvasive Assessment of Liver Fibrosis

For every subject, we collected full patient history (including drugs), anthropometric parameters (BMI), and office blood pressure. Office blood pressure was taken after 5 min rest in the supine position. SBP and DBP were defined according to Korotkoff sounds I and V, respectively.

General biochemistries, which were measured by autoanalyzer, included glucose, glycated hemoglobin (HbA1c), total cholesterol, HDL cholesterol, triglycerides, AST, ALT, GGT, full blood count, ferritin.

Noninvasive assessment of liver fibrosis included both laboratory tests, such as NFS, FIB-4, APRI, as well as imaging tests, such as the point-shear wave elastography. The NAFLD fibrosis score (NFS), which was developed in patients with NAFLD (now called MASLD), was calculated as follows: −1.675 + 0.037 × age (years) + 0.094 × BMI (kg/m^2^) + 1.13 × impaired fasting glycaemia/diabetes (yes = 1, no = 2) + 0.99 × AST/ALT ratio − 0.013 × platelets (10^9^/L) − 0.66 × albumin (g/dL). The fibrosis-4 (FIB-4) index was calculated as follows: [age (years) × AST]/[platelet × ALT^1/2^]. The aspartate aminotransferase (AST) to platelet ratio index (APRI) was calculated as follows: [AST × 100]/platelet].

Liver stiffness measurement was performed with point shear wave elastography (Affinity 70, ElastPQ, Philips Healthcare, Amsterdam, The Netherlands).

The 10-year cardiovascular risk was estimated by the ESC CVD Risk Calculation App, which can be found at https://www.escardio.org/Education/ESC-Prevention-of-CVD-Programme/Risk-assessment/esc-cvd-risk-calculation-app, 16 November 2024. This application includes calculators for primary and secondary prevention in various populations and its use is supported by the 2021 ESC Guidelines on cardiovascular disease prevention in clinical practice [37].

### 4.3. PNPLA3 rs738409 Genotyping

Genotyping of the *PNPLA3* rs738409 polymorphism was performed on peripheral blood mononuclear cells (PBMCs). PBMCs were isolated by density gradient centrifugation from anticoagulant-treated blood samples layered on Ficoll-Paque^TM^ Plus solution (Cytiva, Marlborough, MA, USA). The mononuclear cells obtained were used to extract genomic DNA with the AllPrep DNA/RNA mini kit (Qiagen, Hilden, Germany), following the manufacturer’s instructions. *PNPLA3* rs738409 single nucleotide polymorphism (C > G) was genotyped by real-time TaqMan allelic discrimination assay (TaqMan SNP Assay; ThermoFisher Scientific Waltham, MA USA, ID: C______7241_10). The distribution of allelic frequencies was analyzed by a chi-squared test to show the potential deviation from the Hardy–Weinberg equilibrium. Consistent with previous works [29,30], statistical analyses were performed based on the G allele (GG/CG vs. CC) dominant inheritance pattern.

### 4.4. Arterial Tonometry

As we have recently reported [35], arterial tonometry was performed by a trained operator using the PulsePen® (DiaTecne S.r.l., Milano, Italy). Each patient lied in the supine position. Peripheral blood pressure was measured using a digital sphygmomanometer (OMRON M6 COMFORT HEM-7321-E, OMRON Healthcare, Kyoto, Japan). Pressure waveform calibration was based on the calculation of the mean arterial pressure ((diastolic arterial pressure + peripheral pulse pressure)/3). To measure pulse wave velocity (PWV), i.e., the speed at which the pulse wave runs through the arterial system, we employed the sequential ECG-gated carotid and femoral artery recording method, whereby the tonometer is applied first at the patient’s neck and then at his groin. For more details, see reference [35]. The remaining pulse wave parameters (i.e., cSBP, AI, SEVR, LVET, and SysSlope) were obtained from the carotid pulse wave analysis. Tonometric data were processed by the software WPulsePen (WPP001-ETT–2.3.1; 2013–2019 DiaTecne s.r.l—Italy).

### 4.5. Ultrasound Examination of the Carotid Arteries

Ultrasound examination of the carotid arteries were obtained by high-resolution B-mode ultrasound by a 7.5–12 MHz linear array transducer (Aspen Advanced Ultrasound System, Acuson, Malvern, PA, USA) by a trained operator. Common carotids were examined in posterior-lateral, or medio-lateral directions; longitudinal images of the distal common carotid, in which the interfaces were very clear, were obtained. Carotid mean intima-media thickness (IMT) measurements were performed according to the Mannheim Intima-Media Thickness Consensus [38]. Briefly, the right and left carotid arteries of each subject were examined by the same sonographer with a semiautomatic method. Once an optimal longitudinal image was obtained, it was stored on a 1/2-inch super VHS videotape. Images were analyzed using a high-resolution video recorder, coupled with a mouse-driven image analysis system. IMT, defined as the distance between the lumen–intima and the media–adventitia interfaces, was measured at end diastole in the far wall in each carotid artery segment (common, bulb, internal), bilaterally [9,10]. In each of the above six segments measuring 1 cm of length, mean IMT and maximum IMT were assessed after calibration at the start of the echography study. IMT measurements were expressed as the cumulative mean of mean-IMT (mean-IMT) and as cumulative mean of maximum-IMT (M-MAX) recorded in each vascular segment. In our lab, reproducibility of IMT measurements on separate visits displayed a coefficient of variation of 4.4% and 9.6%, for mean-IMT and maximum-IMT, respectively.

### 4.6. Statistical Analysis

Statistical analysis was performed using the software “R” (version 4.0.3; 2020 The R Foundation for Statistical Computing) and GraphPad Prism (version 8.0.2). A *p* value < 0.05 was considered for statistical significance. The Shapiro–Wilk test was applied to quantitative variables to check for distribution normality. Quantitative variables were reported as the median with interquartile range (IQR); qualitative variables were reported as absolute frequencies and percentages. Univariate correlations were measured with the Pearson or the Spearman test based on data distribution. For continuous variables, two group comparisons were performed with the *t*-test or the Wilcoxon test. Multivariate linear regression was used to explore the effect of patient variables on pulse wave parameters and IMT. Results were reported in terms of the beta regression coefficient with 95% confidence interval.

## 5. Conclusions

MASLD is a heterogeneous entity. For this reason, one study limitation could be the intrinsic difference in the phenotype of metabolic syndrome-driven MASLD vs. genetic MASLD (e.g., age and glucose). Otherwise, limitations of this study include the Mediterranean background of the patients, which might have an impact on genotype distribution [12] as well as lifestyle, and the early stage of MASLD. Nevertheless, this study shows for the first time that *PNPLA3* polymorphism was inversely correlated with aortic stiffness in patients with MASLD without fibrosis. Our data support the view that the main contributors to CVD risk in patients with MASLD remain conventional cardiometabolic risk factors (i.e., age, glucose) that are more likely to be found in metabolic syndrome-related MASLD rather than genetically-driven MASLD, at least in the first stages of the disease. These data might help differentiate patient follow-up, prioritizing CVD assessment in metabolic syndrome-related MASLD. Further studies are needed to confirm and extend our findings to more advanced stages of the disease.

## Figures and Tables

**Table 1:** Characteristics of the entire cohort and *PNPLA3* genotype subgroups.

Variable	All Patients (*n* = 117)	*PNPLA3* CC Genotype (*n* = 37)	*PNPLA3* CG or GG Genotype (*n* = 80)	*p*-Value
Age (years)	63 (55–73)	71 (65–77)	59 (51–70)	0.001
Sex M F	67/117 (57.3%) 50/117 (42.7%)	25/37 (67.6%) 12/37 (32.4%)	42/80 (52.5%) 38/80 (47.5%)	0.12
BMI (Kg/m^2^)	28.5 (26.2–29.5)	27.8 (26.2–31.1)	29.0 (26.3–32.5)	0.35
SBP (mmHg)	135 (120–145)	140 (120–160)	130 (120–140)	0.04
DBP (mmHg)	80 (70–80)	80 (70–80)	80 (70–80)	0.65
Glycaemia (mg/dL)	106 (94–119)	112 (103–121)	102.5 (92–116)	0.04
HbA1c (%)	5.8 (5.5–6.3)	6.0 (5.7–6.6)	5.75 (5.4–6.2)	0.049
HOMA Index	2.58 (2.01–4.13)	2.99 (2.54–4.21)	2.27 (1.65–3.86)	0.08
Diabetes (yes/no)	42/117 (35.9%)	17/37 (45.9%)	25/80 (31.25%)	0.12
Metformin treatment	33/117 (28.2%)	14/37 (37.8%)	19/80 (23.75%)	0.11
GLP-1RA treatment	10/117 (8.55%)	3/37 (8.1%)	7/80 (8.75%)	0.91
SGLT2i treatment	16/117 (13.7%)	4/37 (10.8%)	12/80 (15.0%)	0.54
Total cholesterol (mg/dL)	187 (160–213)	176 (160–207)	193 (161–218)	0.06
HDL (mg/dL)	50 (43–60)	53 (44–58)	50 (43–61)	0.79
LDL (mg/dL)	114.7 (88.7–135.6)	104.2 (70.2–129.8)	118.2 (90.3–137.9)	0.03
Triglycerides (mg/dL)	120 (89–148)	121 (87–137)	119.5 (90.5–156)	0.67
Lipid-lowering therapy (yes/no)	56/101 (55.45%)	21/30 (70.0%)	35/71 (49.3%)	0.06
Creatinine (mg/dL)	0.88 (0.71–1.03)	0.91 (0.81–1.07)	0.83 (0.70–1.01)	0.06
AST (U/L)	24 (20–29)	24 (19–28)	23.5 (20–32)	0.19
ALT (U/L)	25 (19–36)	22 (18–31)	28 (21–37)	0.04
GGT (U/L)	29 (20–44)	28 (19–44)	30 (21–44)	0.62
Ferritin (ug/L)	106.0 (37.2–203.2)	66.6 (34.8–189.0)	110.0 (53.9–211.4)	0.18
NFS (score)	−0.91 (−2.04–0.06)	−0.54 (−1.56–−0.25)	−1.14 (−2.28–0.18)	0.51
FIB-4 (score)	1.35 (0.85–1.78)	1.54 (0.96–1.83)	1.19 (0.81–1.74)	0.16
APRI (score)	0.26 (0.20–0.36).	0.22 (0.18–0.29)	0.26 (0.22–0.37)	0.04
Hepatic stiffness (kPa)	4.71 (4.02–5.73)	4.96 (4.12–5.59)	4.63 (3.98–5.91)	0.99

BMI, body mass index; SBP, systolic blood pressure; DBP, diastolic blood pressure; HbA1c, glycated hemoglobin; HOMA index, Homeostasis Model Assessment index; GLP-1RA, glucagon like peptide-1 receptor agonist; SGLT2i, sodium-glucose co-transporter-2 inhibitors; HDL high-density lipoprotein; LDL, low-density lipoprotein; AST, aspartate transaminase; ALT, alanine transaminase; GGT, gamma glutamyl transferase; NFS, NAFLD fibrosis score; FIB-4, fibrosis-4; APRI, AST to platelet ratio index. Data are presented as median (IQR). Continuous variables were compared with the *t*-test or the Wilcoxon test according to data distribution, while categorical variables were compared with the *Χ*^2^-test. *p* < 0.05 was considered statistically significant.

**Table 2:** Genotype and allele frequencies.

Gene (SNPs)	*n*	Observed Genotype Frequency in Our Cohort			Observed Allele Frequency in Our Cohort		Reported Allele Frequency		*p*
		MM	Mm	mm	M	m	M	m	
*PNPLA3* (rs738409)	117	CC 37 (31.6%)	CG 59 (50.4%)	GG 21 (18.0%)	C 0.57	G 0.43	C 0.79	G 0.21	<0.0001 ^a^ [12]
*PNPLA3* (rs738409)	117	CC 37 (31.6%)	CG 59 (50.4%)	GG 21 (18.0%)	C 0.57	G 0.43	C 0.52	G 0.48	0.328 ^b^ [14]

^a^ Comparison between observed G allele frequency in our cohort and reported G allele frequency in a control Caucasian (European) population; ^b^ Comparison between observed G allele frequency in our cohort and reported G allele frequency in patients with MASLD.

**Table 3:** Vascular features in *PNPLA3* genotype subgroups.

Variable		*PNPLA3* CC Genotype (*n* = 37)	*PNPLA3* CG or GG Genotype (*n* = 80)	*p*-Value
Age (years)		71 (65–77)	59 (51–70)	0.001
Vascular age (years)		78 (69–83)	63 (54–73)	0.001
SBP (mmHg)		136 (125–150)	130 (118–140)	0.039
DBP (mmHg)		81 (75–84)	79 (76–86)	0.842
PP (mmHg)		52 (44–69)	40 (33–56)	0.004
MAP (mmHg)		101 (95–110)	99 (93–106)	0.239
cSBP (mmHg)		133 (117–152)	125 (114–139)	0.029
AI (%)		15.7 (4.5–23.2)	16.5 (5.9–35.4)	0.283
SEVR (%)		100.1 (82.8–113.4)	105.4 (93.1–119.3)	0.250
LVET (ms)		301 (290–308)	306 (282–322)	0.344
SysS (mmHg/ms)		0.92 (0.75–1.07)	0.64 (0.52–0.73)	0.001
PWV (m/sec)		12.2 (10.9–15.2)	9.1 (8.0–11.2)	0.001
IMT mean (mm)		1.00 (0.84–1.07)	0.78 (0.69–0.94)	0.004
MAX IMT (mm)		1.52 (1.31–1.81)	1.30 (1.04–1.77)	0.129
10-year CVD Risk (%)	Low	3.5	13.2	0.001
	Moderate	10.3	37.7	
	High	13.8	20.8	
	Very high	72.4	28.3	

SBP, brachial systolic blood pressure; DBP, brachial diastolic blood pressure; cSBP, central systolic blood pressure; PP, pulse pressure; MAP, mean arterial pressure; AI, augmentation index; SEVR, subendocardial viability ratio; LVET, left ventricular ejection time; SysS, systolic slope; PWV, pulse wave velocity; mean IMT, mean intima-media thickness; MAX IMT, cumulative mean of maximum-IMT. MAP is calculated as DBP + (SBP−DBP)/3). CVD risk estimate is based on the European Society of Cardiology CVD risk calculation App. Data are expressed as median (IQR). Continuous variables were compared with the *t*-test or the Wilcoxon test according to data distribution. *p* < 0.05 was considered statistically significant.

**Table 4:** Multivariate regression analysis on the impact of the genotype of PNPLA3 rs738409 (dominant model) on cSBP, PP, PWV, and mean IMT.

Predictive Variable	β-Estimate	95%CI	*p*-Value
Dependent variable cSBP			
Age	0.33	0.02; 0.63	0.03
Sex [M]	−2.03	−10.35; 6.29	0.63
Glucose	−0.03	−0.20; 0.14	0.71
*PNPLA3* [CG + GG]	−7.36	−16.35; 1.63	0.11
Dependent variable PP			
Age	0.43	0.19; 0.68	<0.001
Sex [M]	−0.29	−7.11; 6.52	0.93
Glucose	−0.05	−0.19; 0.08	0.43
*PNPLA3* [CG + GG]	−6.66	−14.03; 0.70	0.07
Dependent variable PWV			
Age	0.15	0.07; 0.23	<0.001
Sex [M]	1.04	−1.25; 3.37	0.37
Glucose	0.02	−0.03; 0.06	0.50
*PNPLA3* [CG + GG]	−3.04	−5.52; −0.56	0.02
Dependent variable mean IMT			
Age	0.01	0.008; 0.01	<0.001
Sex [M]	0.11	0.02; 0.21	0.02
Glucose	−0.002	−0.004; 0.001	0.08
*PNPLA3* [CG + GG]	0.01	−0.10; 0.12	0.80

cSBP, central systolic blood pressure; PP, pulse pressure; PWV, pulse wave velocity; mean IMT, mean intima-media thickness. CI, confidence interval. *p* < 0.05 was considered statistically significant.

## Data Availability

Raw data that support the findings of this study are available from the corresponding author, upon reasonable request.
